# Investigation on the Short-Term Aging Scheme for High Viscosity Modified Bitumen

**DOI:** 10.3390/ma16113910

**Published:** 2023-05-23

**Authors:** Chengwei Xing, Juze Qin, Zhiqiang Cheng, Mingchen Li, Qingbing Lu

**Affiliations:** 1 Key Laboratory for Special Area Highway Engineering of Ministry of Education, Chang’an University, South 2nd Ring Road Middle Section, Xi’an 710064, China; xingcw@chd.edu.cn; 2 School of Highway, Chang’an University, South 2nd Ring Road Middle Section, Xi’an 710064, China; 3 Changjiang Survey, Planning, Design and Research Co., Ltd., 1863 Jiefang Ave., Wuhan 430010, China; qinjuze@cjwsjy.com.cn; 4 Shanghai Road and Bridge Group Co., Ltd., Shanghai 200433, China; 5 Shanghai Engineering Research Center of Green Pavement Materials, Shanghai 200433, China; 6 The Key Laboratory of Road and Traffic Engineering, Ministry of Education, Tongji University, Shanghai 201800, China

**Keywords:** high viscosity modified bitumen, short-term aging, rolling thin-film oven test, thin-film oven test, aging period, aging temperature

## Abstract

Due to the highly viscous characteristics of high viscosity modified bitumen (HVMB), the commonly used short-term aging schemes are not suitable for it. As such, the objective of this study is to introduce a suitable short-term aging scheme for HVMB by increasing the aging period and temperature. For this purpose, two kinds of commercial HVMB were aged via rolling thin-film oven test (RTFOT) and thin-film oven test (TFOT) at different aging periods and temperatures. At the same time, open-graded friction course (OGFC) mixtures prepared using HVMB were also aged via two aging schemes to simulate the short-term aging of bitumen at the mixing plant. With the aid of temperature sweep, frequency sweep, and multiple stress creep recovery tests, the rheological properties of short-term aged bitumen and the extracted bitumen were tested. By comparing the rheological properties of TFOT- and RTFOT-aged bitumen with those of extracted bitumen, suitable laboratory short-term aging schemes for HVMB were determined. Comparative results showed that aging the OGFC mixture in a 175 °C forced-draft oven for 2 h is suitable to simulate the short-term aging process of bitumen at the mixing plant. Compared with RTOFT, TFOT was more preferable for HVMB. Additionally, the recommended aging period and temperature for TFOT was 5 h and 178 °C, respectively.

## 1. Introduction

With the advancement of the construction of highways, the safety and comfort of driving are being paid increasing attention. In this case, the application scenarios for permeable pavement are becoming more and more popular [1,2,3]. Currently, an open-graded friction course (OGFC) mixture is extensively used for permeable pavement, which is inherently a large air void mixture with air voids of approximately 20% [4,5]. Owing to its large porosity, rainwater can drain away easily through the voids in the mixture, alleviating water logging on the pavement. As a result, it features advantages such as slip resistance, rutting resistance, and noise absorption [6]. However, because of open-graded structures, inadequate adhesion between bitumen and aggregates is a common problem, resulting in pavement damage such as scattering and cracking [7,8]. As such, the bitumen involved in the production of OGFC mixtures needs to be extremely cohesive, with the aggregate to guarantee the performance of the bituminous mixture. Currently, high viscosity modified bitumen (HVMB) is one of the best choices to produce OGFC mixtures [9,10,11], which is a type of modified bitumen that satisfies the basic requirements of normal modified bitumen, as well as satisfies some other requirements such as a dynamic viscosity of over 20,000 Pa.s, a viscous toughness of over 20 N.m, and a toughness of over 15 N.m.

HVMB somewhat solves the problem of poor adhesion of bitumen to aggregates in permeable pavement. Yet, even with the use of HVMB, permeable pavement is still subjected to many damages during its long-term service [12,13,14]. Of these, the most severe pavement damage is the aging of HVMB. The aging of bitumen is a life-cycle problem for bitumen pavement. During the service life, bitumen pavement is subjected to the effects of temperature, oxygen, sunlight, and water, causing the aging of bitumen [15,16,17,18,19]. This will lead to the loss of bitumen’s original properties [20,21,22]. In comparison with conventional dense-graded bitumen pavement, permeable pavement has much larger air voids. That means that bitumen in permeable pavement is more susceptible to the negative effects of sunlight, air, and rain, thereby accelerating the aging process of bitumen [23]. Its accelerated aging makes bitumen pavement vulnerable to cracking, loosening, etc. [24,25]. Thus, the aging behavior of HVMB has been of great interest to researchers [26,27,28,29]. For instance, Sun et al. [26] evaluated the molecular distribution and rheological properties of HVMB before and after long-term oxidative aging using gel permeation chromatography (GPC) and Dynamic shear rheometer (DSR) tests. Based on the reported results, they concluded that during the aging process of HVMB, there were both coupling and parallelism effects that occurred simultaneously. As HVMB aged more heavily, the polymer phase in HVMB continually degraded, and the bitumen phase gradually oxidized. The former caused softening of HVMB, while the latter caused the hardening of bitumen. Hu et al. [27] analyzed the effect of weathering aging on the high-temperature rheological properties and fatigue performance of HVMB. The results of their study showed that, as aging progressed, the high-temperature rutting factor of HVMB gradually increased, while the fatigue life of the bitumen decreased. The magnitude of variation in bitumen’s performance due to weathering aging is correlated with the aging temperature. Conclusively, the long-term aging behavior of HVMB has been studied systematically by researchers via some long-term aging simulation tests, such as pressure aging vessel (PAV) and weathering aging tests.

Whichever protocol is used to simulate the long-term aging process of HVMB, it is required to be preceded by tests to simulate the short-term aging process of bitumen during transport, mixing, and construction. With reference to JTG E20-2011 [30], a rolling thin-film oven test (RTFOT) and thin-film oven test (TFOT) are recommended to simulate the short-term aging process of bitumen. The aging temperature in both tests is 163 °C. The surface of the bitumen specimen in TFOT is stationary, and thus the recommended aging time is long (5 h). In contrast, the surface of the specimen in RTOFT exposed to hot air varies during the test. It is exposed to heat for a shorter period (85 min). These two methods are believed to be useful for simulating the aging of base bitumen plant production. However, a number of studies have shown that these two methods are not suitable for polymer-modified bitumen due to its high viscosity [31,32,33]. Yan et al. [31] tested the properties of SBS-modified bitumen after aging at different RTFOT aging temperatures using DSR and Fourier transform infrared spectroscopy (FTIR). Based on the rheology master curves and carbonyl index of different short-term aged SBS-modified bitumen, they suggested that the RTFOT temperature for bitumen containing 4.5% SBS modifier should be lifted to 178 °C, while bitumen containing 6.0% and 7.5% SBS modifier should be lifted to 193 °C. Similar conclusions were drawn by Xia [33], who proposed that the original temperature and time for the TFOT test should be adjusted to aptly simulate the short-term aging process of SBS-modified bitumen. In summary, it is generally accepted that for polymer-modified bitumen, the time and temperature of short-term aging procedure should be suitably altered. For HVMB, which has a much higher viscosity, it is more problematic to simulate the short-term aging of bitumen at the current aging temperature and time. However, there is still a lack of study on the short-term aging procedure for HVMB.

**Figure 1:**
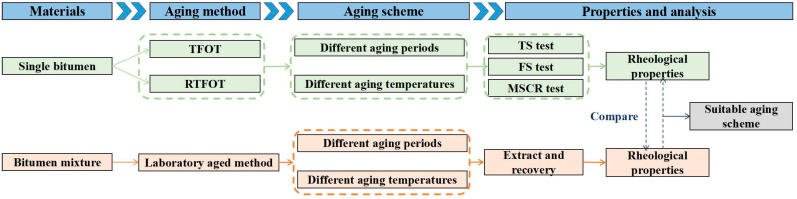
Flow chart of the present study.

Considering the above points, the objective of the present study is to optimize the HVMB short-term aging procedure to simulate the short-term aging of bitumen plant mixing more effectively. For this purpose, two kinds of HVMB were short-term aged at different times and temperatures via TFOT and RTFOT, respectively. Temperature sweep, frequency sweep, and multiple stress creep recovery tests were conducted to analyze the rheological properties of bitumen subjected to different aging procedures. At the same time, the rheological properties of bitumen in laboratory-aged OGFC mixtures were tested after extraction and recovery processes. Finally, the properties of the bitumen extracted from mixtures were compared with bitumen aged via TFOT and RTFOT to propose a suitable short-term aging scheme for HVMB. The flow chart of this study is shown in Figure 1. It is envisaged that the findings of this study may provide assistance to improve the performance of permeable pavements. 

## 2. Materials and Methods

First, an overview of the materials and methods used in this study is given. For the reader’s convenience, Table 1 summarizes all the acronyms used in this study.

### 2.1. Materials

In this study, two kinds of commercial high viscosity modified bitumen were used, which are composed of bitumen and high viscosity modifier. The former is a mixture of hydrocarbons. Additionally, the latter is primarily a composition of thermoplastic rubber, resin, anti-aging agents, and plasticizers. The difference between the two HVMBs lies in the different proportions of each component added to the modifier. The basic properties of these two HVMBs are listed in Table 2. Basalt and limestone were selected as aggregates to prepare OGFC-13 mixtures, which have a maximum nominal particle size of 13.2 mm. Among them, the coarse aggregate was basalt and the fine aggregate and mineral fillers were limestone. To produce the loose OGFC mixture for the short-term aging test, the HVMB was first mixed with aggregate at 185 °C for 90 s, followed by an additional mixing process with mineral fillers for another 90 s.

### 2.2. Methods

#### 2.2.1. Short-Term Aging Procedure

In this study, the short-term aging process of HVMB was simulated via RTFOT and TFOT. Table 3 shows the differences between these two aging methods. As mentioned above, it is quite difficult to successfully simulate the aging process of HVMB during plant mixing using the standard RTFOT and TFOT, since the viscosity of HVMB is so high. Two schemes were trialed in this study to address this issue. The first scheme was to alter the aging period in TFOT and RTFOT. For TFOT, four aging periods were used, including 3, 5, 7, and 9 h. For RTFOT, the aging periods were set as 55, 75, 95, and 115 min. The aging temperature was first set as 163 °C, just the same as the standard procedure. The second scheme was to alter the aging temperature in TFOT and RTFOT. Here, three aging temperatures were selected, including 163, 178, and 193 °C, and the aging period was determined based on the results of the first aging scheme.

**Figure 2:**
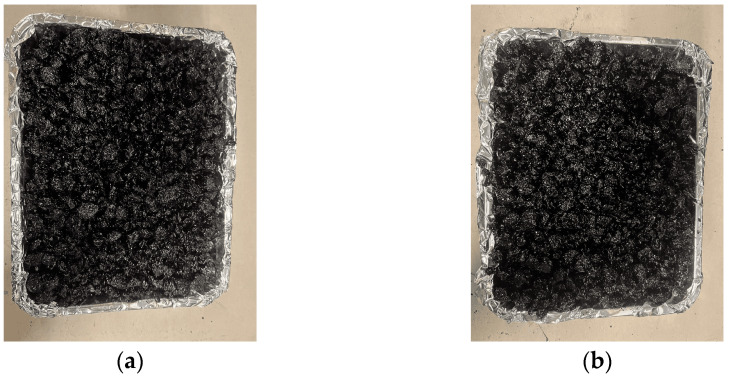
Short-term aged OGFC mixtures prepared with: (**a**) HVMB-A; (**b**) HVMB-B.

For comparison, the OGFC bitumen mixtures were equally subjected to the short-term aging scheme mixture to simulate short-term aging at the mixing plant. In this study, two short-term aging schemes for bituminous mixtures recommended in AASHTO R 30-02 [34] were adopted. For the first aging scheme, the loose bitumen mixture was placed evenly in a pan, ensuring that its thickness ranged from 25 to 50 mm, as shown in Figure 2. Then, it was put in a 175 °C forced-draft oven and aged for 2 h. The mixture in the pan was turned over with a shovel at hourly intervals to ensure uniform aging. As for the second scheme, it was the same as the first aging scheme except that the aging temperature was decreased to 135 °C and the aging time was increased to 4 h. After the mixtures were short-term aged, the bitumen was recovered from the mixtures via the extraction and recovery method [35,36]. Following this, the properties of bitumen aged via TFOT and RTFOT were compared with the bitumen extracted from mixture to determine the proper bitumen short-term aging scheme. Table 4 shows the different aging schemes and their corresponding identifications.

#### 2.2.2. Temperature Sweep (TS) Test

TS test was conducted based on AASHTO T315 [37] in this study to obtain the complex modulus(G*) and phase angle of HVMB under different aging conditions at 58 °C, 64 °C, 70 °C, and 76 °C. The samples of bitumen were positioned between two parallel plates, which were subjected to a sinusoidal oscillation load at 10 rad/s.

#### 2.2.3. Frequency Sweep (FS) Test

FS test of HVMB under different aging conditions was conducted to construct the master curve of HVMB according to AASHTO TP5-93 [38]. It is generally confirmed that the master curve of bitumen describes the linear viscoelastic rheological behavior of bitumen over a range of frequencies at the given temperature. In this study, frequency sweep tests were conducted via strain control mode in the range of 0.1 to 30 Hz, covering temperatures from 15 to 75 °C. To ensure that all bitumen samples were within the linear viscoelastic range across all frequencies and temperatures, a strain level of 0.15% was selected for the 15 to 25 °C test and a strain level of 1.5% was selected for the 35 to 75 °C test. Based on the results of frequency tests, the master curve of bitumen’s complex modulus was constructed with reference to the Generalized Logistic Sigmoidal model [39].

#### 2.2.4. Multiple Stress Creep Recovery (MSCR) Test

In this study, MSCR test was conducted on HVMB under different aging conditions in accordance with ASSHTO TP-350 [40]. During the test, the bitumen samples were first subjected to 20 creep/recovery cycles at the stress of 0.1 kPa, followed by 10 creep/recovery cycles at the stress of 3.2 kPa. Each creep/recovery cycle included 1 s of creep procedure and 9 s of recovery procedure. The mean percent recovery for 10 creep/recovery cycles at two stress levels was yielded using Equation (1), and the mean non-recoverable creep compliance for 10 creep/recovery cycles at two stress levels was calculated using Equation (2). Notably, the data for the 11th to 20th creep/recovery cycles under 0.1 kPa stress level were used for the calculation of *R*_0.1_ and *J_nr_*_0.1_.

(1) $$R=\frac{\varepsilon_{p}-\varepsilon_{u}}{\varepsilon_{p}}$$

(2) $$J_{nr}=\frac{\varepsilon_{u}}{\sigma }$$

where *R* refers to the percent recovery, %; *J_nr_* refers to non-recoverable creep compliance, kPa^−1^; $\varepsilon_{p}$ refers to peak strain, %; $\varepsilon_{u}$ refers to unrecovered strain, %; and σ refers to the applied shear stress, kPa.

## 3. Results and Discussion

### 3.1. Effect of Aging Period on Short-Term Aging Scheme

In this study, an attempt was first made to propose a new short-term aging scheme to realistically simulate the short-term aging process of HVMB during the mixing and construction process in plants by modifying the aging periods of RTFOT and TFOT. To judge the suitability of the altered aging scheme, the rheological properties of RTFOT- and TFOT-aged bitumen were compared with the bitumen extracted from short-term aged mixtures. The specific rheological characteristics included complex modulus, phase angle, master curve, *R*, and *J_nr_*.

#### 3.1.1. TS Test

**Figure 3:**
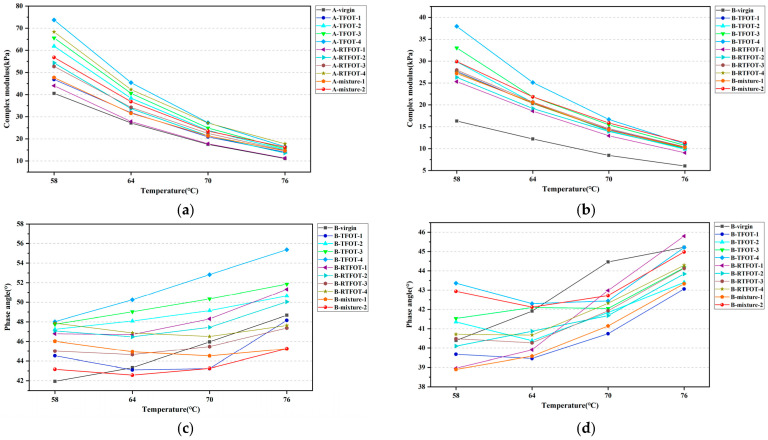
TS tests results: (**a**) Complex modulus of HVMB-A; (**b**) Complex modulus of HVMB-B; (**c**) Phase angle of HVMB-A; (**d**) Phase angle of HVMB-B.

The complex modulus and phase angle of the aged bitumen were obtained using TS tests, and results are shown in Figure 3. As can be seen from the figure, the complex modulus of two kinds of HVMB exhibited a downward trend as the temperature increased. It was due to the increase in temperature, which intensified the irregular movement of the bitumen molecules, resulting in a reduction in the stress required for the same strain response. Therefore, the bitumen was less able to resist the action of external forces, as manifested by a decrease in the modulus of the bitumen. For both RTFOT and TFOT aging methods, raising the period of short-term aging resulted in an increase in the complex modulus of two kinds of modified bitumen. Regarding HVMB-A, the complex modulus of bitumen in the A-mixture-1 and A-mixture-2 roughly matched that of bitumen aged via TFOT for 3 to 5 h or that aged using RTFOT for 55 to 75 min. For HVMB-B, the complex modulus of bitumen in these two short-term aged mixtures was approximately equivalent to that of the bitumen aged using TFOT for 5 to 7 h. However, using RTFOT, the complex modulus of short-term aged HVMB-B was still lower than that of the bitumen in the mixture subjected to 135 and 175 °C short-term aged processes. It suggests that for HVMB-B, the RTFOT method is not a satisfactory simulation of the short-term aging of bitumen in the mixture during the mixing and paving process. The reason for this is that HVMB, which is highly viscous, does not fully melt and spread evenly into a uniform film at that temperature (163 °C). As a result, the surface of the specimen that is exposed to hot air during RTFOT remains unchanged throughout the test, resulting in the insufficient aging of high viscosity modified bitumen.

Regarding the phase angle of HVMB, the phase angle of two kinds of HVMB does not show a regular change with increasing aging periods in both aging approaches. This occurs owing to the fact that degradation of the modifier phase and aging of the bitumen phase occur simultaneously during the short-term aging process [41]. The former will decrease the elasticity of bitumen, whereas the latter has the opposite effect. The evolution of the phase angle of HVMB after short-term aging depends on the competition between the aging rates of bitumen and modifier phase. For HVMB-A, the phase angle of bitumen extracted from the mixtures aged via two methods approximately corresponded to bitumen aged with TFOT for 3 h. In contrast, the phase angle of RTFOT-aged bitumen differed considerably with those extracted from mixtures. With respect to HVMB-B, the phase angle of bitumen in the mixture aged at 135 °C for 4 h corresponded to that of bitumen that underwent between 5 and 7 h of TFOT. The phase angle of bitumen in the mixture aged at 175 °C for 2 h roughly matched that of bitumen subjected to a 9 h TFOT test. Similarly, RTFOT failed to simulate the variations in phase angle of bitumen during the short-term process of the mixtures very well.

#### 3.1.2. FS Test

**Figure 4:**
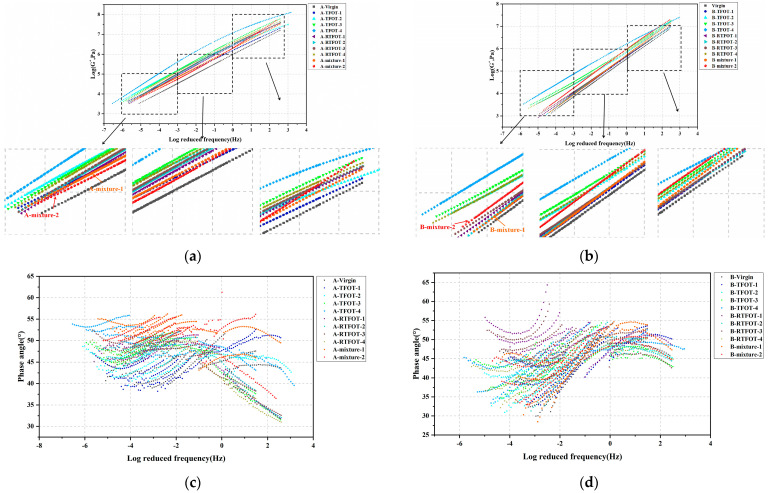
FS tests results: (**a**) Complex modulus master curve of HVMB-A; (**b**) Complex modulus master curve of HVMB-B; (**c**) Phase angle of HVMB-A; (**d**) Phase angle of HVMB-B.

FS tests were conducted for the prepared bitumen specimen; based on the obtained results, complex modulus master curves of the bitumen specimen were constructed using a Generalized Logistic Sigmoidal model. Of note is that the phase angle master curve for polymer-modified bitumen is usually constructed using a Double Logistic model. However, the viscoelastic rheological behavior of HVMB used in this study is complex, and the accuracy of the fit is poor when using a Double Logistic model to construct the phase angle master curve. Considering the above points, the phase angle variations at different temperatures and frequencies are only listed without constructing their master different bitumen specimen yielded in FS test. Figure 4 presents the complex modulus master curve and phase angle curve of two kinds of HVMB. Notably, the modulus master curve of bitumen contains the modulus of the bitumen at many frequencies, so it is difficult to determine the most appropriate method of bitumen aging from a single point value. As such, the master curve of bitumen was divided into three regions, including low-, medium-, and high-frequency regions. The similarity of the modulus master curves in the low-, medium-, and high-frequency regions of bitumen prepared under different aging schemes and the modulus master curve of extracted bitumen from the mixtures were compared to find the appropriate short-term aging scheme.

It can be seen from the figure that regardless of the short-term aging method, the complex modulus of HVMB at different frequencies increases with the increasing aging periods. For HVMB-A, the complex modulus master curves of bitumen extracted from two mixtures almost overlapped. In the low- and medium-frequency regions of the master curve, the complex modulus of bitumen in the mixture roughly corresponded to that of bitumen after 3 h of TFOT aging. In the high-frequency regions, the complex modulus of bitumen in mixtures roughly matched that of bitumen after 5 to 7 h of TFOT. For HVMB-B, the complex modulus of bitumen extracted from the mixtures subjected to 135 °C aging for 4 h approximately corresponded to that of bitumen aged with 3 h TFOT or 75 min RTFOT in low- and medium-frequency regions. Additionally, it roughly matched that of bitumen aged via 7 h TFOT or 115 min RTFOT in the high-frequency region. The complex modulus of bitumen in the mixtures subjected to 175 °C aging for 2 h generally matched that of bitumen aged via 5 h of TFOT or 95 to 115 min RTFOT in the low- and medium-frequency regions. In the high-frequency region, the complex modulus of bitumen in the above mixture matched that of bitumen that suffered 7 to 9 h of TFOT. In comparison with TFOT, altering the aging period of RTFOT had a small effect on the complex modulus master curve of HVMB, and TFOT can better simulate the variation of bitumen’s complex modulus master curve during the short-term aging process of OGFC mixtures. 

In terms of phase angles of HVMB at different-frequencies, since the existing master curve fitting models were not applicable for the HVMBs selected in this study, it is difficult to find a suitable short-term aged method via comparing the phase angle of bitumen at different frequencies. Subsequent studies will consider alternative fitting models to construct phase angle master curves with a view of analyzing the feasibility of short-term aging scenarios by comparing phase angle master curves of HVMB.

#### 3.1.3. MSCR Test 

**Figure 5:**
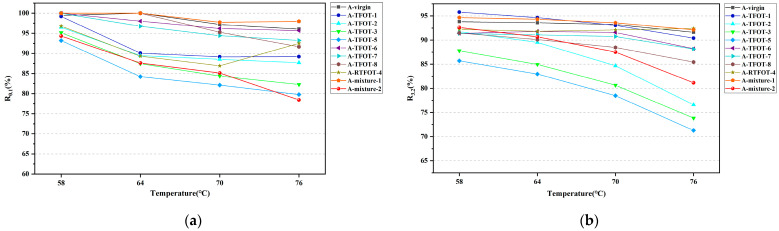

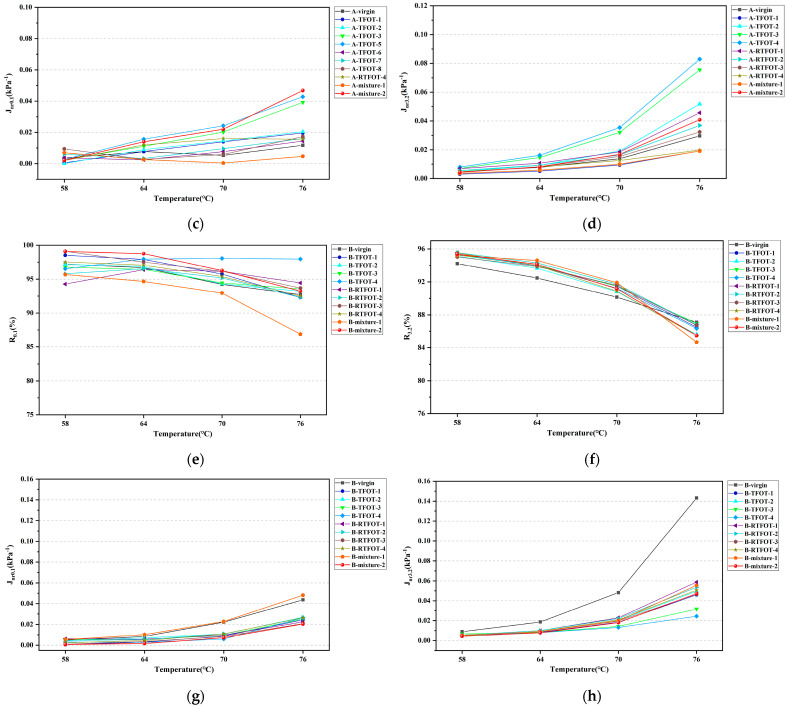
MSCR results: (**a**) *R*_0.1_ of HVMB-A; (**b**) *R*_3.2_ of HVMB-A; (**c**) *J_nr_*_0.1_ of HVMB-A; (**d**) *J_nr_*_3.2_ of HVMB-A; (**e**) *R*_0.1_ of HVMB-B; (**f**) *R*_3.2_ of HVMB-B; (**g**) *J_nr_*_0.1_ of HVMB-B; (**h**) *J_nr_*_3.2_ of HVMB-B.

MSCR tests were performed on two kinds of HVMB with varying aging conditions. Figure 5a–h shows the calculated *R*_0.1_, *R*_3.2_, *J_nr_*_0.1_, and *J_nr_*_3.2_ of the bitumen with varying aging conditions. As can be seen from the figure, two high viscosity modified bitumen present quite different behavior in terms of percent recovery and non-recoverable creep compliance after short-term aging. For HVMB-A, the *R*_0.1_, *R*_3.2_, *J_nr_*_0.1_, and *J_nr_*_3.2_ of HVMB-A after different periods of RTFOT were closer to the virgin bitumen and the change was not significant. In contrast, as the TFOT aging periods increased, the percent recovery at two stress levels of bitumen gradually decreased, and the non-recoverable creep compliance increased. This conclusion differs from the regular evolution of base bitumen after short-term aging. The reason for this is that for HVMB, in addition to the aging of bitumen phase, the degradation of the modifier also occurs during the short-term aging process [41]. The combined effect of these two determines the change in percent recovery and non-recoverable creep compliance of bitumen after short-term aging. For HVMB-B, less change in bitumen’s percent recovery and non-recoverable creep compliance was found after RTFOT and TFOT. The difference in the effect of short-term aging method and period on the percent recovery and non-recoverable creep compliance of two HVMBs may be explained by the different types and dosages of modifiers.

Based on the MSCR quantification results for two kinds of HVMB and bitumen extracted from the mixtures, the correlation between the aging degree for RTFOT- and TFOT-aged bitumen and that of bitumen extracted from mixtures was compared. Results are shown in Table 5. It can be concluded from the table that for HVMB-A, the aging degree of bitumen extracted from the mixture aged at 135 °C for 4 h roughly matches that of virgin bitumen. In comparison with RTFOT, bitumen aged via TFOT provides a better simulation of bitumen aging behavior in the mixture aged at 175 °C for 4 h. However, comparing different indicators gives different optimal aging periods in TFOT. For HVMB-B, whatever aging method or period was selected, the MSCR results of aged bitumen aged via TFOT and RTFOT were close to those extracted from the short-term aged mixture.

#### 3.1.4. Optimum Aging Method Period Analysis

In view of the above reported results, it can be concluded that in comparison with RTFOT, TFOT can better simulate the short-term aging process of HVMB that occurs during the mixing process of the mixtures. This is because the viscosity of HVMB is so high that the fluidity of bitumen is affected, which significantly compromises the aging effect of RTFOT. Using TFOT, the short-term aging process of HVMB in the mixture during the mixing process can be reasonably simulated. However, the optimum aging periods for TFOT still need to be discussed. Table 6 summarizes the suitable TFOT aging periods obtained in TS, FS, and MSCR tests. It can be seen from the table that it is difficult to give an exact short-term aging period for TFOT based on the available results that is suitable for all kinds of HVMB. In summary, the aging degree of bitumen extracted from the mixture aged at 135 °C roughly matched that of bitumen after 3 to 5 h of TFOT. The aging degree of bitumen extracted from the mixture aged at 175 °C approximately corresponded to that of bitumen that underwent 5 to 7 h of TFOT aging. The aging degree of bitumen in the mixtures aged with these two schemes is not the same. When the mixture was aged at 135 °C, there was little change in the rheological properties of bitumen in the mixture after aging. Even some of the rheological properties of aged bitumen aged at 135 °C were identical to those of virgin bitumen. This is because 135 °C aging temperature is more suitable for base bitumen. It may be low for HVMB, hence bitumen aging is not evident. For HVMB, the mixing, transport, and paving temperature of the mixture is much higher than that of base bitumen. In this case, we consider that aging the mixture at 175 °C, which is determined via the compacting temperature of the high viscosity modified bitumen, is a closer way of simulating the short-term aging process of high viscosity modified bitumen mixtures during its mixing, transport, and paving processes. As such, its corresponding 5 to 7 h are considered as suitable aging periods in TFOT to simulate the short-term aging process of HVMB.

### 3.2. Effect of Aging Temperature on Short-Term Aging Scheme

In addition to aging period, the effect of aging temperature on short-term aging procedure was evaluated in this study. Considering the above findings, only TFOT was adopted to simulate the short-term aging process of bitumen mixture during the mixing process. In addition to the conventional 163 °C, two additional aging temperatures (178 and 193 °C) were selected. Based on the above findings, the aging periods were set as 5 and 7 h. Table 7 presents the supplementary aging schemes and their corresponding identifications.

#### 3.2.1. TS Test

**Figure 6:**
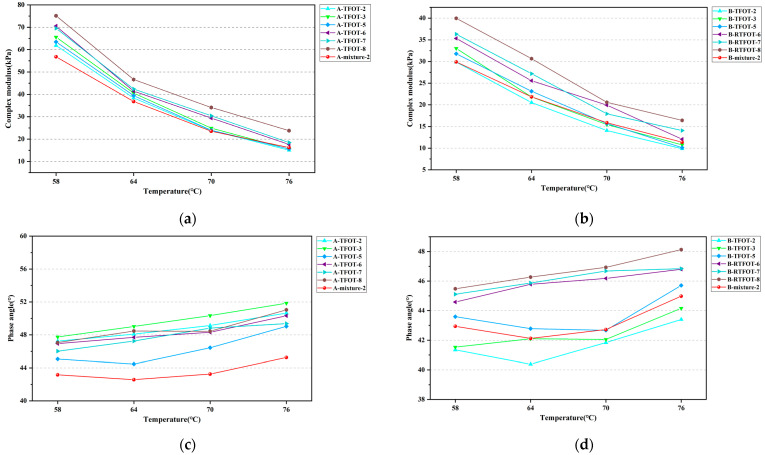
TS tests results of bitumen aged at different temperatures: (**a**) Complex modulus of HVMB-A; (**b**) Complex modulus of HVMB-B; (**c**) Phase angle of HVMB-A; (**d**) Phase angle of HVMB-B.

The complex modulus and phase angle of bitumen aged at different aging temperatures were tested using a TS test; results are shown in Figure 6. It can be seen from the figure that as the aging temperature of TFOT rose and the complex modulus of two kinds of HVMB increased, indicating that more severe aging of bitumen occurs at this time. In contrast, the effect of increasing aging temperature on the phase angle of these two kinds bitumen was not the same. The elevation of the aging temperature caused a gradual increase in the phase angle of HVMB-B, but the phase angle of HVMB-A showed irregular changes. The difference may be explained by the different types and dosages of modifiers used in these two bitumen. Based on the complex modulus and phase angle of bitumen obtained via TS test, it can be achieved that the aging degree of bitumen in the mixtures is closest to that of the bitumen aged at 178 °C for 5 h in TFOT.

#### 3.2.2. FS Test

**Figure 7:**
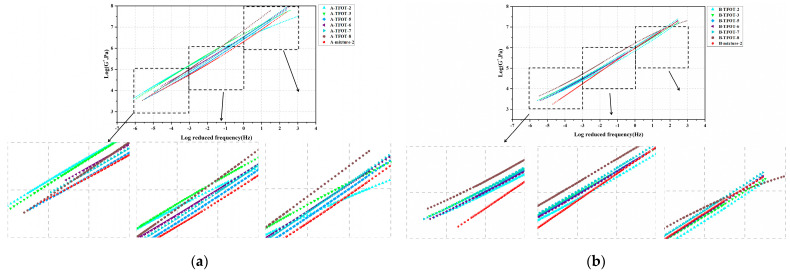
FS tests results of bitumen aged at different temperatures: (**a**) Complex modulus master curve of HVMB-A; (**b**) Complex modulus master curve of HVMB-B.

An FS test was further conducted for the bitumen aged at different temperatures. Additionally, considering the above results, only the master curve of complex modulus was constructed via a Generalized Logistic Sigmoidal model; results are shown in Figure 7. It can be derived from the figure that the effect of aging temperature on the complex modulus master curve of two HVMBs was not the same. For HVMB-A, as the aging temperature increased, the complex modulus of HVMB decreased in the low-frequency region while increasing in the high-frequency region. In contrast, it showed an opposite variation law in the low-frequency region for HVMB-B. For HVMB-A, the master curve of bitumen extracted from short-term aged mixtures was closest to that of bitumen aged at 178 °C for 5 h. For HVMB-B, in addition to bitumen aged at 193 °C for 7 h, the master curves of bitumen aged at other schemes were all similar to those of bitumen extracted from short-term aged mixtures.

#### 3.2.3. MSCR Test

**Figure 8:**
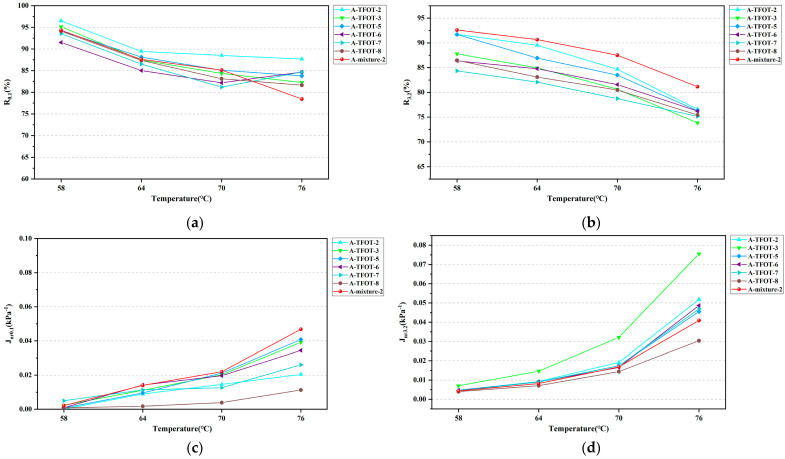

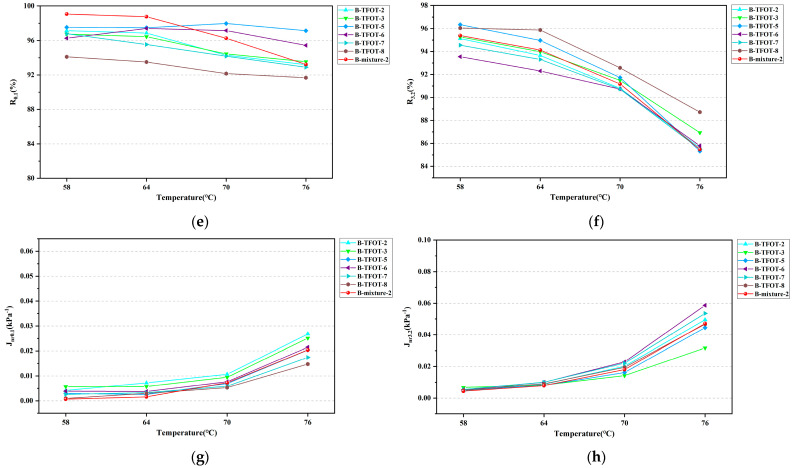
MSCR tests results of bitumen aged at different temperatures: (**a**) *R*_0.1_ of HVMB-A; (**b**) *R*_3.2_ of HVMB-A; (**c**) *J_nr_*_0.1_ of HVMB-A; (**d**) *J_nr_*_3.2_ of HVMB-A; (**e**) *R*_0.1_ of HVMB-B; (**f**) *R*_3.2_ of HVMB-B; (**g**) *J_nr_*_0.1_ of HVMB-B; (**h**) *J_nr_*_3.2_ of HVMB-B.

An MSCR test was further conducted for the bitumen aged at different temperatures. Additionally, the *R*_0.1_, *R*_3.2_, *J_nr_*_0.1_, and *J_nr_*_3.2_ of HVMB were calculated; results are shown in Figure 8. It can be concluded from the figure that, as the aging temperature of TFOT increased, the MSCR quantification results of two HVMBs showed no regular variation. By comparing the geometric similarity of aged and extracted bitumen curves, the suitable aging periods and temperatures in TFOT were determined; results are shown in Table 8. As can be seen from the figure, aged HVMB at 178 for 5 h in TFOT is a suitable aging scheme. The percent recovery and non-recoverable creep compliance of the bitumen extracted from the mixture and bitumen aged via this scheme are relatively close.

#### 3.2.4. Optimum Aging Method Period and Temperature Analysis

In view of the above-reported results, it is suggested that raising the aging temperature of TFOT is also one of the solutions to the problem that existing aging schemes are not suitable for HVMB. It seems more effective to raise the aging temperature than extend the aging period. A comprehensive comparison of TS, FS, and MSCR results shows that, in contrast to other aging schemes, the aging degree of bitumen in the short-term mixtures closely matches that of bitumen aged at 178 °C for 5 h in TFOT. Furthermore, to investigate whether this short-term aging scheme can better simulate the short-term aging process of HVMB in the on-site mixtures; the bias of the rheological properties of bitumen aged via the proposed short-term aging scheme and recovered bitumen from the mixture were calculated; results are shown in Table 9. It can be seen from the table that, except the *J_nr_*_0.1_ of the bitumen at 58 and 64 °C, other rheological properties of HVMB after short-term aging with the scheme proposed in this study were closer to those of extracted bitumen from the short-term aged mixtures. The deviation was within an acceptable 10%. For *J_nr_*_0.1_ of the bitumen at 58 and 64 °C, it was close to 0 for both TFOT-aged bitumen and extracted bitumen. Therefore, the relative deviation obtained from the calculation appears to be large. Essentially, the real deviation between the two was still small. Conclusively, the proposed aging scheme is applicable to simulate the short-term aging process of HVMB in the on-site mixtures. It will be further validated for its generalizability across more types of HVMB.

## 4. Conclusions

With the aid of TS, FS, and MSCR tests, the effect of short-term aging schemes on the aging condition of HVMB was investigated in this study. Based on the reported findings, a suitable short-term aging method for HVMB was proposed. With the aid of the proposed aging scheme, the aging level of HVMB in the short-term aging mixtures can be well-simulated. The main conclusions are summarized as follows:

(1) After a short-term aging process, the complex modulus of HVMB measured via TS test gradually increases, but no regular variations are observed for other rheological properties. The aging behavior of HVMB is more complicated, involving degradation of the modifier and aging of the bituminous phase. The combination of these two factors ultimately determines the change in bitumen properties after short-term aging.
(2) Compared with RTFOT, TFOT offers a better simulation of the short-term aging process of HVMB in the mixing plant. For TFOT, 5 h and 178 °C are recommended as a suitable period and temperature to simulate the short-term aging process of HVMB.
(3) Aging the HVMB mixtures at 135 °C for 4 h results in no noticeable change in the rheological properties of HVMB. In contrast, aging the HVMB mixture at 175 °C for 2 h can better simulate the short-term aging process of HVMB in the on-site mixtures.

In this study, only two kinds of HVMBs were adopted to investigate the short-term aging schemes applicable to HVMB. The generalizability of the determined temperature and period will be subsequently tested with more kinds of HVMB. If possible, subsequent studies will also take HVMB mixture samples from construction sites to verify the reliability of the HVMB mixture’s short-term aging scheme recommended in this study.

## Figures and Tables

**Table 1:** Acronyms used in this study.

Acronym	Full Description
OGFC	Open-graded friction course
HVMB	High viscosity modified bitumen
RTFOT	Rolling thin-film oven test
TFOT	Thin-film oven test
TS	Temperature sweep
FS	Frequency sweep
MSCR	Multiple stress creep recovery

**Table 2:** Basic properties of two HVMBs.

Property	HVMB-A	HVMB-B	Test Method
Penetration (25 °C, 100 g, 5 s, 0.1 mm)	35	49	ASTM D5
Softening point (°C)	92	87	ASTM D36
Ductility (5 cm/min, cm)	52.2	47.3	ASTM D113
Dynamic viscosity (60 °C, Pa.s)	>400,000	>50,000	ASTM D2171
Rotary viscosity (165 °C, Pa.s)	2.15	1.89	ASTM D4402

**Table 3:** Difference between TFOT and RTFOT.

Difference	TFOT	RTFOT
Sample container	Flat-bottomed plate	Glass bottle
Sample thickness	About 3.2 mm	5~10 μm
Rotational speed/(r.min^−1^)	5.5 ± 1	15 ± 0.2
Bituminous film motion state	Relatively static	Continuous movement

**Table 4:** Aging scheme and identifications of selected bitumen.

Bitumen Type	Aging Method	Aging Temperature (°C)	Aging Period (Minutes)	Identification
HVMB-A	Virgin	-	-	A-virgin
	TFOT	163	180	A-TFOT-1
			300	A-TFOT-2
			420	A-TFOT-3
			540	A-TFOT-4
	RTFOT	163	55	A-RTFOT-1
			75	A-RTFOT-2
			95	A-RTFOT-3
			115	A-RTFOT-4
	Short-term aged for mixture	135	240	A-mixture-1
		175	120	A-mixture-2
HVMB-B	Virgin	-	-	B-virgin
	TFOT	163	180	B-TFOT-1
			300	B-TFOT-2
			420	B-TFOT-3
			540	B-TFOT-4
	RTFOT	163	55	B-RTFOT-1
			75	B-RTFOT-2
			95	B-RTFOT-3
			115	B-RTFOT-4
	Short-term aged for mixture	135	240	B-mixture-1
		175	120	B-mixture-2

**Table 5:** Aging degree of bitumen extracted from mixture corresponded to that of bitumen aged by RTFOT and TFOT.

Indicator	Bitumen Type	Properties of Bitumen in Mixture Aged at 135 °C for 4 h		Properties of Bitumen in Mixture Aged at 175 °C for 2 h	
		Aging Period in TFOT (Hours)	Aging Period in RTFOT (Minutes)	Aging Period in TFOT (Hours)	Aging Period in RTFOT (Minutes)
*R*_0.1_	HVMB-A	0 (virgin)	0 (virgin)	5 to 7	More than 115
	HVMB-B	All	All	All	All
*R*_3.2_	HVMB-A	0 to 3	0 to 55	3 to 5	95
	HVMB-B	All	All	All	All
*J_nr_*_0.1_	HVMB-A	0 (virgin)	0 (virgin)	7	More than 115
	HVMB-B	0 (virgin)	0 (virgin)	All	All
*J_nr_*_3.2_	HVMB-A	0 (virgin)	0 (virgin)	3 to 5	75 to 95
	HVMB-B	All	All	All	All

**Table 6:** Suitable TFOT aging period obtained by TS, FS, and MSCR tests.

Indicator	Bitumen Type	Suitable Aging Periods to Simulate the Aging Behavior of Bitumen in the Mixture after 135 °C Aging				Suitable Aging Periods to Simulate the Aging Behavior of Bitumen in the Mixture after 175 °C Aging			
		3 h	5 h	7 h	9 h	3 h	5 h	7 h	9 h
Complex modulus (TS)	HVMB-A	√	√			√	√		
	HVMB-B		√	√			√	√	
Phase angle (TS)	HVMB-A	√					√	√	
	HVMB-B	√							√
Complex modulus (low- and medium-frequency)	HVMB-A	√				√			
	HVMB-B	√					√		
Complex modulus (high-frequency)	HVMB-A		√	√			√	√	
	HVMB-B		√					√	√
*R*_0.1_(MSCR)	HVMB-A	-	-	-	-	-	√	√	-
	HVMB-B	√	√	√	√	√	√	√	√
*R*_3.2_(MSCR)	HVMB-A	√				√	√		
	HVMB-B	√	√	√	√	√	√	√	√
*J_nr_*_0.1_(MSCR)	HVMB-A	-	-	-	-			√	
	HVMB-B	-	-	-	-	√	√	√	√
*J_nr_*_3.2_(MSCR)	HVMB-A	-	-	-	-	√	√		
	HVMB-B	√	√	√	√	√	√	√	√

**Table 7:** Aging scheme and identifications of selected bitumen.

Aging Method	Bitumen Type	Aging Temperature (°C)	Aging Period (Hours)	Identification
TFOT	HVMB-A	178	5	A-TFOT-5
			7	A-TFOT-6
		193	5	A-TFOT-7
			7	A-TFOT-8
	HVMB-B	178	5	B-TFOT-5
			7	B-TFOT-6
		193	5	B-TFOT-7
			7	B-TFOT-8

**Table 8:** Suitable aging scheme in TFOT obtained via MSCR.

Indicator	Bitumen Type	163 °C 5 h	163 °C 7 h	178 °C 5 h	178 °C 7 h	193 °C 5 h	193 °C 7 h
*R*_0.1_	HVMB-A		√	√		√	√
	HVMB-B	√	√	√	√	√	
*R*_3.2_	HVMB-A	√		√			
	HVMB-B	√	√	√		√	
*J_nr_*_0.1_	HVMB-A		√	√	√		
	HVMB-B		√	√		√	√
*J_nr_*_3.2_	HVMB-A	√		√	√	√	√
	HVMB-B	√	√	√	√	√	√

**Table 9:** Deviation between properties between bitumen aged via the proposed scheme and extracted bitumen from short-term aged bitumen mixture.

Rheological Properties		Deviation between Bitumen and Extracted Bitumen from the Mixture	
		HVMB-A	HVMB-B
Complex modulus (kPa)	58 °C	10.4%	5.8%
	64 °C	6.6%	5.5%
	70 °C	1.9%	−1.1%
	76 °C	−3.9%	11.2%
Phase angle (°)	58 °C	4.2%	1.5%
	64 °C	4.3%	1.5%
	70 °C	6.8%	0.1%
	76 °C	7.7%	1.6%
*R*_0.1_	58 °C	0.1%	1.6%
	64 °C	0.6%	1.3%
	70 °C	0.0%	1.7%
	76 °C	6.3%	4.0%
*R*_3.2_	58 °C	1.0%	0.9%
	64 °C	4.3%	0.9%
	70 °C	4.7%	0.6%
	76 °C	6.4%	0.2%
*J_nr_*_0.1_	58 °C	−177%	70.1%
	64 °C	44.6%	27.1%
	70 °C	3.6%	−4.9%
	76 °C	10.7%	0.0%
*J_nr_*_3.2_	58 °C	3.9%	12.9%
	64 °C	9.3%	1.7%
	70 °C	3.6%	−11.0%
	76 °C	10.0%	−6.0%

## Data Availability

The data presented in this study are available on request from the corresponding author.
